# Self-knotting catheter post urethroplasty: a case report

**DOI:** 10.1016/j.eucr.2026.103420

**Published:** 2026-03-24

**Authors:** Ronald Sugianto, Soetojo Wirjopranoto, Dimas Panca Andhika

**Affiliations:** a Department of Urology, Faculty of Medicine, Airlangga University, Surabaya, Indonesia; b Dr. Soetomo General Academic Hospital, Surabaya, Indonesia; c Department of Anatomy, Histology, and Pharmacology, Faculty of Medicine, Airlangga University, Surabaya, Indonesia; d Universitas Airlangga Hospital, Surabaya, Indonesia

**Keywords:** Malfunction catheter, Percutaneous cystoscopy, Knotting catheter

## Abstract

Intravesical catheter knotting is a rare and challenging complication. This case report aims to report the management of a self-knotted catheter following urethroplasty in a pediatric patient. We report a 6-year-old male patient, 14 days after urethroplasty, who complained of urinary retention and a stuck catheter. Abdominopelvic x-ray and urology ultrasonography showed a suspicion of material surrounding the catheter. Transcutaneous cystoscopy showed a knotting catheter. Removal of the tied catheter segment resulted in the successful removal of the urethral catheter. Even though knotting catheters is a rare complication, it is a potential cause of failed catheter removal.

## Highlights

• Intravesical catheter knotting is a rare and challenging complication.
• The predisposing factor is disproportionate to the catheter used in the bladder.
• The removal techniques for knotting catheters are various.
• Transcutaneous cystoscopy is a safe technique for diagnostic and therapeutic.

## 1 Introduction

Urethroplasty is a commonly performed surgery for hypospadias. Hypospadias management goals focus on curvature correction and adequate diameter of neo-urethral to prevent urinary obstruction, cosmetics, and preserve ejaculation function.^1^ The urinary catheter post-urethroplasty is commonly placed to prevent urine extravasation to the surrounding tissues, which can disturb tissue healing.^2^ However, it imposes a significant burden during the postoperative period, such as discomfort, inactivity, urinary tract infection, and even postoperative urethral stricture.^3^

The duration of catheter placement is still debatable; immediate removal post-procedure may reduce discomfort feelings and shorten the length of stay, but it is associated with a higher incidence of wound dehiscence or urethro-cutaneous fistula due to disruption of the healing tissue. Conversely, a prolonged indwelling catheter is associated with a higher risk of infection, which leads to urethral stricture or meatal stenosis.^1^ A rare yet challenging complication is intravesical catheter knotting. Although uncommon, several cases have been reported.^4^ Therefore, we report and describe the prevention and management of this complication. This manuscript was prepared following the CARE guidelines (https://www.care-statement.org).^5^

## 2 Case presentation

**Fig. 1:**
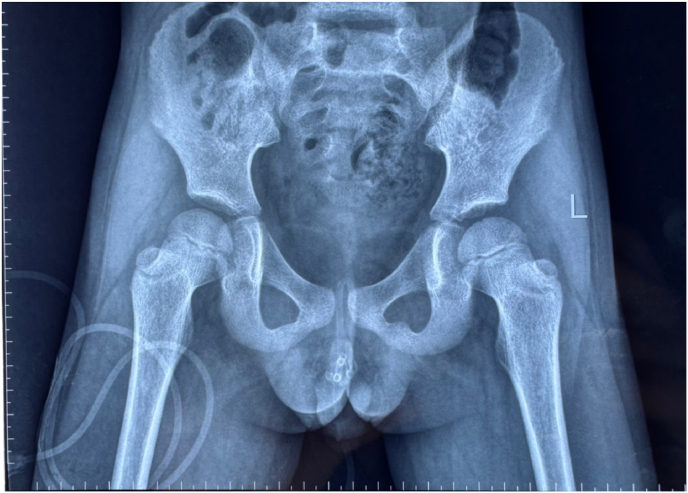
BNO showed the suspicion of encrusted catheter.

A 6-year-old male patient had a hypospadias that underwent the chordectomy and urethroplasty. The patient was inserted with a non-ballooning catheter for 10 days to maintain the neourethral lumen. On the scheduled catheter removal date, the catheter could not be removed easily. The catheter got stuck and malfunctioned, and the patient had urinary retention. Abdominopelvic x-ray showed a suspicion of material surrounding the catheter, as shown in Fig. 1. Therefore, the patient was diagnosed with a stuck catheter and planned for transcutaneous cystoscopy.

**Fig. 2:**
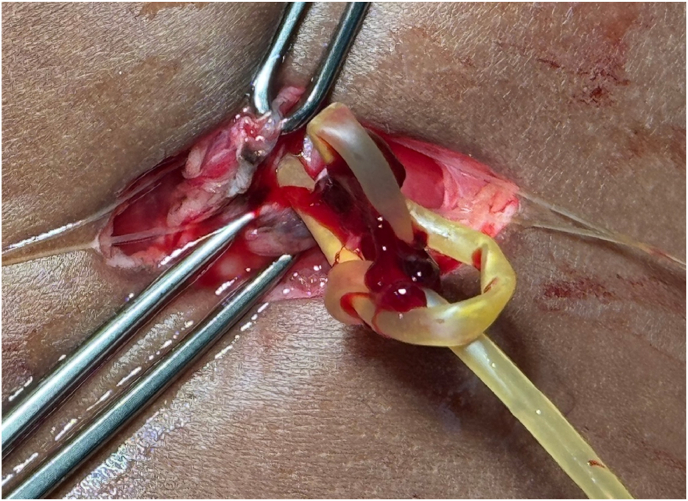
Intravesical knotting of the catheter.

**Fig. 3:**
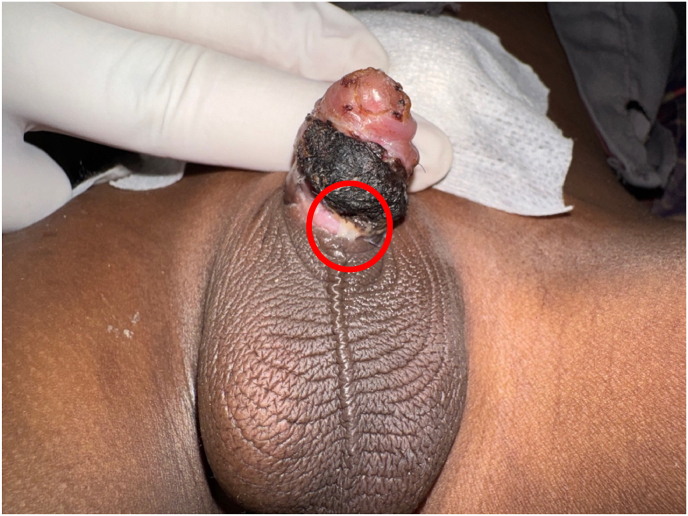
Urethro-cutaneous fistula as a complication of a malfunctioning catheter.

During the cystoscopy, it was found that the catheter had formed a knot, as shown in Fig. 2. The tied catheter segment was removed transcutaneously with an aligator, and then the rest of the catheter segment in the urethra can be removed easily. The urethral catheter and cystostomy catheter used a 12 French balloon silicone catheter for urinary drainage. Five days after the operation, the urethral catheter was removed to evaluate the urinary stream, but the patient showed multiple urethrocutaneous fistulae at the proximal penile urethra, as shown in Fig. 3. Therefore, six months later, the patient was planned for redo urethroplasty to repair his urethra.

## 3 Discussion

Knotting of urethral catheters is a rare complication of urinary catheterization, especially when feeding tubes are used for urinary drainage.^5^ The main predisposing factors in the pediatric population are disproportionate to the catheter used in the bladder, such as small caliber catheters, small bladder capacity, excessive length of catheter insertion, catheter softness and flexibility, and bladder spasm that induces intravesical coiling.^6^ If a flexible catheter is inserted excessively into the bladder, a loop may form spontaneously, or the catheter tip may migrate through the coil, resulting in a knot when the catheter is removed.^5^^,^^7^

The removal techniques for knotting catheters are various, including systemic steroid application, manual extraction, guiding wire insertion in the catheter lumen, or surgical management.^7^ Systemic steroid treatment reduces urethral edema, increasing the likelihood of successful manual extraction.^8^ Guiding wire–assisted fluoroscopy aims to increase the rigidity to straighten and untie the knot.^9^ The last management is a surgical approach, which can be suprapubic open cystostomy and urethro-cystoscopy.^6^^,^^10^ In our case, we used transcutaneous cystoscopy to evaluate the cause of the malfunctioning catheter.

To prevent the knotting of urinary catheters, we recommend (i) careful catheter selection, avoiding overly flexible feeding tubes, (ii) estimating the length of neo-urethra and normal proximal urethra according to pediatric age to safe margin insertion lengths, (iii) avoiding prolonged indwelling time and improper insertion technique, and (iv) Gently removing the catheter to minimize the damaging internal structures or healing tissue.^11^ We consider that the limitation of this report is that it only performs a minimal diagnostic procedure. However, the strength of our study is the first case that happened after urethroplasty, which was indwelling for relatively short periods.

## 4 Conclusion

Transcutaneous cystoscopy provides a safe diagnostic and therapeutic approach for malfunctioning or knotting catheters. Postoperative follow-up should assess the complications in the neo-urethra as a consequence of knotting catheters.

## CRediT authorship contribution statement

**Ronald Sugianto:** Writing – review & editing, Writing – original draft, Validation, Investigation, Formal analysis, Data curation, Conceptualization. **Soetojo Wirjopranoto:** Writing – review & editing, Validation, Supervision, Investigation. **Dimas Panca Andhika:** Writing – review & editing, Validation, Supervision, Investigation, Data curation.

## Informed consent statement

Informed consent for the publication of this case report and the accompanying images was obtained from the patient's parents in the Indonesian language. The parents acknowledged understanding and voluntarily agreed to the publication anonymously. A signed copy of the written consent has been securely stored. This document is available for review upon request by the corresponding author.

## Ethical clearance

This research has received approval from the research ethics committee of the Faculty of Medicine, Universitas Airlangga, Soetomo General Academic Hospital with No. 4124/109/3/XII/2025.

## Funding statement

None.

## Conflict of interest

No conflict of interest.
